# Identification of different trypanosome species in the mid-guts of tsetse flies of the Malanga (Kimpese) sleeping sickness focus of the Democratic Republic of Congo

**DOI:** 10.1186/1756-3305-5-201

**Published:** 2012-09-19

**Authors:** Gustave Simo, Barberine Silatsa, Njiokou Flobert, Pascal Lutumba, Philemon Mansinsa, Joule Madinga, Emile Manzambi, Reginald De Deken, Tazoacha Asonganyi

**Affiliations:** 1 Department of Biochemistry, Faculty of Science, University of Dschang, PO Box 67, Dschang, Cameroon; 2 Faculty of Science, University of Yaoundé 1, Yaoundé , Cameroon; 3 Department of Tropical Medicine, School of Medicine, Kinshasa University, Kinshasa, Democratic Republic of Congo; 4 Programme National de Lutte contre la Trypanosomiase Humaine Africaine, Kinshasa, Democratic Republic of the Congo; 5 Institut National de Recherche Biomédicale, Kinshasa, Democratic Republic of the Congo; 6 Department of Animal Health, Institute of Tropical Medicine, Nationalestraat 155, 2000, Antwerp, Belgium; 7 Faculty of Medicine and Biomedical Sciences, University of Yaoundé 1, Yaounde, Cameroon

**Keywords:** Sleeping sickness, Animal African trypanosomiasis, Trypanosomes, Blood meals, Tsetse flies, Mid-guts

## Abstract

**Background:**

The Malanga sleeping sickness focus of the Democratic Republic of Congo has shown an epidemic evolution of disease during the last century. However, following case detection and treatment, the prevalence of the disease decreased considerably. No active survey has been undertaken in this focus for a couple of years. To understand the current epidemiological status of sleeping sickness as well as the animal African trypanosomiasis in the Malanga focus, we undertook the identification of tsetse blood meals as well as different trypanosome species in flies trapped in this focus.

**Methods:**

Pyramidal traps were use to trap tsetse flies. All flies caught were identified and live flies were dissected and their mid-guts collected. Fly mid-gut was used for the molecular identification of the blood meal source, as well as for the presence of different trypanosome species.

**Results:**

About 949 *Glossina palpalis palpalis* were trapped; 296 (31.2%) of which were dissected, 60 (20.3%) blood meals collected and 57 (19.3%) trypanosome infections identified. The infection rates were 13.4%, 5.1%, 3.5% and 0.4% for *Trypanosoma congolense* savannah type, *Trypanosoma brucei* s.l., *Trypanosoma congolense* forest type and *Trypanosoma vivax*, respectively. Three mixed infections including *Trypanosoma brucei* s.l. and *Trypanosoma congolense* savannah type, and one mixed infection of *Trypanosoma vivax* and *Trypanosoma congolense* savannah type were identified*.* Eleven *Trypanosoma brucei gambiense* infections were identified; indicating an active circulation of this trypanosome subspecies. Of all the identified blood meals, about 58.3% were identified as being taken on pigs, while 33.3% and 8.3% were from man and other mammals, respectively.

**Conclusion:**

The presence of *Trypanosoma brucei* in tsetse mid-guts associated with human blood meals is indicative of an active transmission of this parasite between tsetse and man. The considerable number of pig blood meals combined with the circulation of *Trypanosoma brucei gambiense* in this focus suggests a transmission cycle involving humans and domestic animals and could hamper eradication strategies. The various species of trypanosomes identified in the Malanga sleeping sickness focus indicates the coexistence of animal and human African Trypanosomiasis. The development of new strategies integrating control measures for human and animal trypanosomiasis may enable the reduction of the control costs in this locality.

## Background

Sleeping sickness or Human African trypanosomiasis (HAT) is a fatal disease that occurs only in sub-Saharan Africa. About 60 million persons are exposed to the risk of infection with about 10,000 new cases reported per year [1]. The disease cycle includes three components: the trypanosome (*Trypanosoma brucei gambiense* or *Trypanosoma brucei rhodesiense*), the tsetse fly (*Glossina*), and the host (human or animal). HAT occurs generally in rural areas, and during the end of the 20^th^ century, many outbreaks in historical foci were observed [2]. Faced with these outbreaks, considerable efforts were deployed to fight against this disease. This resulted in a considerable decrease in transmission rate and a consequential decrease in surveillance.

The keystones of interventions against HAT are active and passive case finding, followed by treatment, vector control, and animal reservoir management. However, in most affected countries, case finding followed by treatment are the cornerstones of the disease control. This strategy was shown to permit a reduction in the number of reported new cases by about 69% during the period from 1997–2006 [3]. Following this success, the elimination of the disease was declared. To avoid the re-emergence of the disease, as was the case in 1960; control and surveillance of the disease must not be abandoned. In fact, in 1960 when HAT was under control in most endemic countries, the advent of independence in most of the countries led to a decline in disease awareness, due to the rarity of HAT cases, and because the newly independent authorities had other priorities. This has resulted in the resurgence of the disease in most historical foci. This resurgence was probably due to the presence of undiagnosed patients during case detection, an increase in the population of infected flies, which can transmit the parasite to healthy people, and to the presence of animal reservoirs from which tsetse flies can get infected.

The current epidemiological context of HAT is similar to the situation 50 years ago. This suggests that if there is no improvement of surveillance strategies, there may be a decline in awareness of the disease, since most endemic countries are faced with new challenges like HIV/AIDS, malaria, tuberculosis. The consequence will be a slow return of the disease over time, and there will be a resurgence of the disease in most former endemic areas. To avoid such an eventuality, there is need to introduce new control and surveillance strategies involving new measures, since elimination of HAT as a public health problem requires continuous efforts and innovative approaches. The transmission cycle shows tsetse flies as the central actors that transmit the parasite to vertebrate hosts. Therefore, the integration of tsetse flies as an important component of new control or surveillance strategies is crucial if the disease is to be eliminated. For example, the identification of *T. b. gambiense* in the tsetse fly is an indication that the transmission of the parasite occurs in the disease focus. In addition, the identification of infected flies and the localization of areas where tsetse flies take most of their blood meals may enable areas of high disease transmission to be defined. In the past, such approaches were difficult to undertake due to difficulties in identifying *T. b. gambiense* in tsetse flies, and identifying various tsetse fly vertebrate hosts. During the last decades, molecular tools have been developed to identify blood meals [4,5] and different trypanosome species in tsetse flies and mammals [6,7]. For instance, the identification of trypanosomes in mammals and vectors has been considerably improved by the PCR-based methods [6,8-12]. These tools provided data that enabled the understanding of the disease transmission cycle as well as the epidemiology of HAT.

In the present work, we aimed to identify the origin of blood meals and the trypanosome species present in the mid-guts of tsetse flies caught in the Malanga HAT focus of the Bas Congo Province of the Democratic Republic of Congo, in order to understand the current epidemiological situation of the disease and to generate data enabling an understanding of animal trypanosomosis in this locality.

## Methods

### Study zone

**Figure 1:**
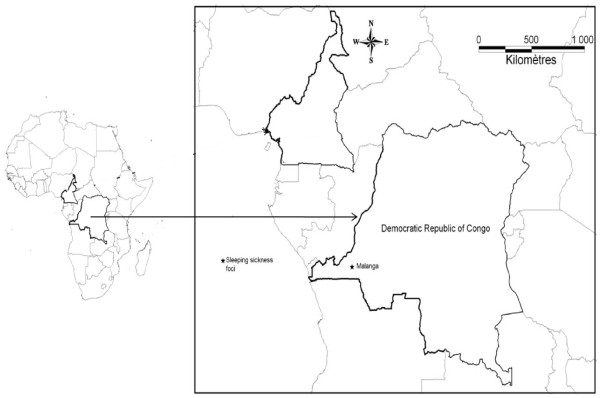
Map showing the geographic location of the Malanga sleeping sickness focus in the Democratic Republic of Congo.

The Malanga (4°34’S, 14°21’E) HAT focus is located in the Bas Congo Province of the Democratic Republic of Congo (Figure 1). This focus lies along the Lukunga River and has vegetation that is characterized by the presence of herbaceous savannah and forest relics. The Malanga focus is situated at 232 km from Kinshasa and 133 km from Matadi. Its altitude is estimated at 340 meters. The inhabitants of this area mostly practice subsistence farming and small scale animal husbandry. Farms situated along valleys and domestic animals such as goats, sheep and pigs are found in this focus, most of which move freely around the village. *Glossina palpalis palpalis* is the only tsetse fly species found in this focus. No vector control activities have been deployed in this area for several years. During the last decade, the Malanga HAT focus was among the most active foci in the Democratic Republic of Congo (DRC). However, during the last couple of years the epidemiological situation in this focus has not been examined.

### Entomologic survey

**Figure 2:**
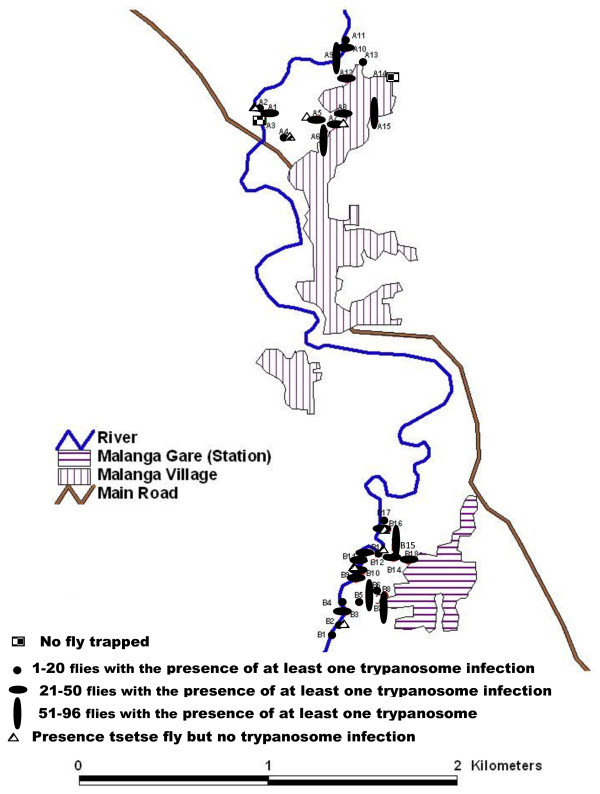
Capture sites presenting the localization of the traps with the range of trapped flies per trap as well as the traps where trypanosome infections were identified.

The entomological survey was performed in August 2009 during the dry season. During this survey, 33 pyramidal traps [13] were deployed for 4 consecutive days in two capture sites (“Malanga gare” and Malanga village) of the Malanga HAT focus (Figure 2). For each trap, the geographical coordinates were recorded using a Global Positioning System (GPS). Tsetse flies were collected twice a day, counted, and the species and sex of each of them determined. Living flies were dissected and the mid-gut extracted. In general, tsetse flies were dissected the same day. However, in instances where all flies could not be dissected on the same day of collection, they were wrapped up in wet floor cloth and dissected early the next morning before the arrival of the first round of collection. After dissection, the presence of residual blood meals in tsetse mid-gut was recorded. The mid-guts were collected into micro-tubes containing ethanol and maintained in the field at about 25 °C. In the laboratory, the tubes were kept at -20 °C until the extraction of DNA.

### DNA extraction

In the laboratory, the alcohol in the micro-tubes was evaporated for 60 minutes in an oven set at 80°C. Subsequently, 300 μl of Chelex 5% was added to each tube and the mixture vortexed for 10 minutes. Thereafter, the tubes were incubated first at 56 °C for 30 minutes, and then at 98 °C for 60 minutes. The tubes were centrifuged at 14 000 rpm for 5 minutes and the supernatant (DNA extract) was collected and stored at −20 °C until used.

### Identification of the blood meal origins

Blood meals were identified using Heteroduplex PCR assay as described by Njiokou et al. [4]. Briefly, the amplification of cytochrome B gene was performed in 25 μl of a mixture containing 5 μl of DNA extract and 20 pmol of each vertebrate cytochrome B primers (Cyt B1/2) [5]. From the amplified DNA fragments, heteroduplexes were formed by hybridization with Giant rat cytochrome B DNA chosen as a driver [4]. The heteroduplex DNA profiles were resolved on 5% acrylamide/urea gel as described by Njiokou et al. [4]. The blood meal origin was identified by comparing the heteroduplex profiles of each blood meal with those of reference vertebrate hosts commonly found in this HAT focus like man and domestic animals (pig, goat, and sheep).

### Identification of different species of trypanosomes

For this identification, two approaches were used: the first approach focused on the internal transcribed spacer 1 (ITS) of ribosomal DNA as described by Desquesnes et al. [14], whereas the second approach used primers specific to *Trypanozoon*[15], *T. congolense* forest type [15], *T. congolense* savannah type [15], *T. vivax*[15] and *T. simiae*[15].

For the ITS, the amplification reactions were carried out as described by Farikou et al. [16]. Briefly, two pairs of primers designed for ribosomal DNA were used. The first amplification round was performed in a final volume of 25 μl containing 2 μl of DNA extract (template), 20 picomoles of each primer (TRYP18.2C: 5’-GCAAATTGCCCAATGTCG-3’ TRYP4R: 5’-GCTGCGTTCTTCAACGAA-3’), 200 mM of each dNTP and 0.5 unit of Taq DNA polymerase. One denaturing step at 94 °C for 3 minutes was followed by 30 amplification cycles. Each cycle consisted of a denaturation step at 94 °C for 30 seconds, an annealing step at 51 °C for 30 seconds, and an extension step at 72 °C for 2 minutes and a final extension at 72 °C for 10 minutes. The amplified products were diluted 10 fold and 2 μl of each dilution was used as a template for the nested PCR. This latter was performed using primers IRFCC (5’CCTGCAGCTGGATCAT 3’) and TRYP5RCG (5’ATCGCGACACGTTGTG 3’). The conditions and the amplification program for the nested PCR were identical to those described for the first PCR. Then micro-liters of the amplified products of the nested PCR were resolved on 2% agarose gel containing 10 mg/μl of ethidium bromide. The gels were observed on ultraviolet light and photographed.

For the second approach which uses primers specific to *Trypanozoon*[15], *T. congolense* forest type [15], *T. congolense* savannah type [15], *T. vivax*[15] and *T. simiae*[15], the amplification conditions were as described previously [17].

**Figure 3:**
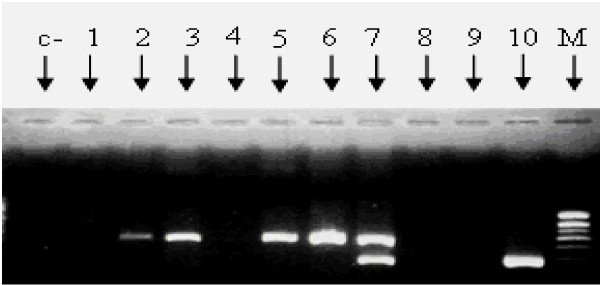
**Example of agarose gel showing the resolution of the amplified products of ITS-1 of different trypanosome species.** M: Molecular marker; C- : negative control; 1, 4, 8 and 9: samples showing no trypanosome infection; 2: *T. congolense* positive control; 10: *T. brucei* s.l. positive control; 5 and 6; samples showing *T. congolense* infections; 7: samples showing mixed infections of *T. brucei* s.l. and *T. congolense.*

To identify *T. b. gambiense,* all samples with a multi-copy 177 bp repeat sequence specific for trypanosomes of the subgenus *Trypanozoon* or for which a DNA fragment of a molecular weight corresponding to that of *T. brucei* (Figure 3) were selected. From these samples, a second PCR was performed to search for the presence of *T. b. gambiense*. This second PCR was carried out as described by Simo et al. [17] using TRBPA1/2 primers that amplify an allele of 149 bp characteristic of group 1 *T. b. gambiense*[18]. All samples that did not shown the allele of 149 bp through the second PCR were considered here as being infected by *T. b. brucei,* since the other species (*Trypanosoma evansi* and *Trypanosoma brucei rhodesiense*) of the subgenus *Trypanozoon* are probably absent in the region due to their geographical localization. Since *T. brucei* s.l. is a diploid organism, a mixed infection of *T. b. brucei* and *T. b. gambiense* was identified when TRBPA1/2 primers showed more than two alleles.

## Results

### Entomological survey

During the entomological survey, 33 pyramidal traps were deployed and 949 *Glossina palpalis palpalis* were trapped (Table 1): 563 females and 386 males. Two hundred and ninety six tsetse flies (31.2%) were dissected and 42 teneral flies (fly which had never taken a blood meal) identified. About 70% of trapped flies died in the trap or during the period between their capture and the dissection. Most traps deployed had at least one tsetse fly during the four days of capture. The range of tsetse flies caught per trap is reported in Figure 2. The average apparent density of tsetse flies per day was 7.2 for both sites and 7.3 for site A and 6.82 for site B (Table 1). Among the dissected flies, 60 (6.33% of the overall trapped flies) blood meals were collected.

**Table 1:** Results of the entomological survey and the identification of blood meals as well as different species of trypanosomes according to each trap

**Trap number**	**Latitude**	**Longitude**	**Flies Trapped**	**Dissected flies**	**ADP**	**Blood meals**	**Teneral**	**TB**	**TBG**	**TV**	**TCS**	**TCF**
Site A (Malanga village)												
A1	5.54660°	14.34885°	23	12	5.75	-	3	-	-	-	-	1
A2	5.54633°	14.34854°	14	3	3.5	-	1	-	-	-	-	-
A3	5.54692°	14.34871°	-	-	-	-	-	-	-	-	-	-
A4	5.54774°	14.34972°	5	-	1.25	-	-	-	-	-	-	-
A5	5.54687°	14.35132°	32	6	8	-	1	-	-	-	-	-
A6	5.54742°	14.35162°	96	17	24	2HB, 2UN	2	-	-	-	2	-
A7	5.54710°	14.35212°	33	5	8.25	-	-	-	-	-	-	-
A8	5.54658°	14.35253°	35	10	8.75	2 PB	3	-	-	-	2	-
A9	5.54418°	14.35217°	71	9	17.75	2 PB, 1HB	4	1	1	-	-	1
A10	5.54348°	14.35270°	47	11	11.75	4 PB, 1HB	1	1	1	-	3	2
A11	5.54320°	14.35263°	18	3	4.5	1 PB	-	-	-	-	1	-
A12	5.54494°	14.35270°	28	9	7	3 PB	2	-	-	-	3	-
A13	5.54412°	14.35347°	5	3	1.25	-	-	1	1	-	-	-
A14	5.54497°	14.35431°	-	-	-	-	-	-	-	-	-	-
A15	5.54694°	14.35401°	51	10	12.75	-	3	-	-	-	1	-
Total			458	98	7.3	12 PB, 4HB, 2UN	20	3	3	0	12	4
Site B (Malanga gare)												
B1	5.57142°	14.35199°	7	6	1.75	1UN	2	1	1	-	-	-
B2	5.57081°	14.35233°	9	2	2.25	1 PB	-	-	-	-	-	-
B3	5.57023°	14.35267°	25	15	6.25	3HB	1	-	-	1	1	1
B4	5.56977°	14.35247°	18	9	4.5	2 PB	-	1	1	-	-	1
B5	5.56973°	14.35330°	6	7	1.5	1HB	-	1	1	-	1	-
B6	5.56927°	14.35414°	13	7	3.25	3 PB	-	-	-	-	1	-
B7	5.56967°	14.35420°	59	17	14.75	6HB	1	3	3	-	3	1
B8	5.56944°	14.35452°	75	22	18.75	4 PB, 1HB	3	-	-	-	2	1
B9	5.56864°	14.35313°	40	3	10	3HB	1	1	1	-	-	-
B10	5.56828°	14.35343°	24	8	6	-	2	-	-	-	-	-
B11	5.56776°	14.35339°	26	15	6.5	-	1	1	1	-	1	-
B12	5.56758°	14.35390°	23	17	5.75	2 PB	3	-	-	-	1	-
B13	5.56748°	14.35422°	7	3	1.75	-	1	-	-	-	-	-
B14	5.56768°	14.35495°	21	15	5.25	2 PB	4	-	-	-	2	-
B15	5.56720°	14.35477°	59	12	14.75	2UN	2	1	-	-	2	-
B16	5.56635°	14.35462°	21	9	5.25	3 PB	1	-	-	-	-	-
B17	5.56583°	14.35449°	9	2	2.25	-	-	1	-	-	1	-
B18	5.56777°	14.35561°	49	30	12.25	6 PB, 2HB	1	-	-	-	7	1
Total			491	198	6.82	23 PB, 16HB, 3 UN	22	10	8	1	22	5

*HB*: Blood meals taken on humans; *PG*: Blood meals taken on pigs; *UN*: blood meals not identified despite the amplification of cytochrome B gene; *ADP*: apparent density of tsetse flies per trap per day; *TB*: *Trypanosoma brucei* s.l.; *TBG*: *Trypanosoma brucei gambiense*; *TCF*: *Trypanosoma congolense* forest type; *TCS*: *Trypanosoma congolense* savannah type; *TV*: *Trypanosoma vivax.*

### Blood meal identification

Of the 60 blood meals sampled, the origin of 55 (91.7%) of them was successfully identified. In addition to using human, domestic animals (pig, goat, sheep) commonly found in this HAT focus, 15 wild animal species were also used as references [19]. Five (8.3%) blood meals could not be identified because their heteroduplex profiles did not correspond to those of the vertebrate hosts used as references. Of the 55 blood meals successfully identified, 35 (58.3%) were from pigs, 20 (33.3%) from man and 5 (8.3%) from other mammals not yet identified (Table 1).

### Identification of trypanosome species

Out of 296 tsetse flies dissected, the identification of trypanosomes was performed on 254 mid-guts because 42 were teneral flies. Of the 254 mid-guts analyzed, the amplification of internal transcribed spacers and the specific primers to *Trypanozoon*, *T. congolense* forest and *T. congolense* savannah type enabled us to identify 57 (22.4%) mid-guts infected by at least one trypanosome species (whatever the species). Among these 57 infections, 75.4% (43/57) were due to trypanosomes of the subgenus *Nannomonas*. No *T. simiae* infection was identified either by internal transcribed spacer or *T. simiae* specific primers. Looking at the distribution of trypanosomes per trap, it appears that most traps caught tsetse flies infected by at least one species of trypanosome (Figure 2).

Of the 57 infections revealed by both methods (ITS of rDNA and species specific PCR), the ITS revealed 48 (20.25%) mid-guts infected by different trypanosome species or subspecies, including one *Duttonella* (*T. vivax*), 4 *Trypanozoon* and 43 *Nannomonas* (*T. congolense* forest and savannah types). The 43 *Nannomonas* infections included 9 (21%) *T. congolense* forest type and 34 (79%) *T. congolense* savannah type. Four mixed infections involving different trypanosome species were identified (Figure 3): three mixed infections of *Trypanozoon* and *T. congolense* savannah type, and one mixed infection of *T. vivax* and *T. congolense* savannah type*.*

The use of *Trypanozoon* specific primers, which target a multi-copy 177 bp repeat sequence revealed 13 (5.1%) mid-guts infected with *T. brucei* s.l. The four *T. brucei* mid-gut infections identified by ITS of rDNA method were confirmed by the *Trypanozoon* specific primers. Moreover, nine additional *T. brucei* mid-gut infections were identified by the second method. The number of *T. brucei* infections revealed by the *Trypanozoon* specific primers was more than 3 times the number of infections revealed by ITS.

The TRBPA primers [18] that amplified an allele of 149 bp characteristic of group 1 *T. b. gambiense,* revealed 11 (4.3%: 11/254) mid-guts infected with this trypanosome subspecies among the 13 *Trypanozoon* positive samples. Moreover, 6 mixed infections involving *T. b. gambiense* and *T. b. brucei* were identified.

Of the 254 tsetse flies dissected, 13.4% (34/254) were infected with *T. congolense* savannah type, 3.5% (9/254) with *T. congolense* forest type, 0.4% (1/254) with *T. vivax* and 5.1% (13/254) with *Trypanozoon*; 4.3% (11/254) with *T. b. gambiense* and 3.14% (8/254) with *T. b. brucei*.

## Discussion

To improve our knowledge on the epidemiological status of HAT and AAT in the Malanga sleeping sickness focus, tsetse flies were trapped and the blood meal origins, as well as different species of trypanosomes, were identified in tsetse mid-guts. *Glossina palpalis palpalis* was the only tsetse fly species trapped in this focus, meaning that it is the main vector of the disease in this area. The high percentage of dead flies could be due to the high temperature observed during the dry season. In addition, the higher ADP may be another explanation. For instance, the average ADP observed during this study was about 7; indicating that more than 231 tsetse flies were trapped per day. This considerable number of flies trapped per day was difficult to dissect on the same day and some flies died overnight despite the fact that the tubes containing these flies were wrapped up in wet floor cloth.

The percentage (6.33%) of residual blood meals obtained here is higher than the values obtained (2.8% and 4.7%; [20] and [16], respectively) for the same tsetse species in southern Cameroon. The difference can be explained by the composition of the fauna as well as the number of domestic and wild animal species found in each focus. In the forest HAT focus of southern Cameroon where different wild animal species are found [19,21], very few domestic animals (especially pigs) are bred. Therefore, tsetse flies may have difficulty taking blood meals due to the vivacity of wild animals compared to pigs and other domestic animals. In the Malanga focus where considerable numbers of pigs are found, wild animals are rare [22]. Tsetse flies could easily take blood meals on domestic animals like pigs. This hypothesis is strengthened by the high percentage (58.3%) of pig blood meals found in tsetse flies from this focus. In the Malanga HAT focus, humans appeared as the second source of blood meals for tsetse flies, since about 33% of the blood meals were taken on humans; illustrating thus an important human/tsetse contact in this focus. Moreover, the identification of *T. b. gambiense* in the mid-gut of tsetse flies of this focus strengthens these human/tsetse contacts and suggests an active transmission HAT in the Malanga focus. This also suggests a transmission cycle including human and tsetse flies.

The results of the identification of different species of trypanosomes seem to indicate that the species-specific PCR method is more sensitive than the ITS, since 13 *Trypanozoon* infections were identified by specific PCR, whereas, only 4 of these infections were identified by ITS. These results are in line with those obtained by Desquenes et al. [14] who showed that a specific PCR method was more sensitive than the ITS in identifying different trypanosome species in African livestock. Using the ITS, different trypanosome species can be revealed with only two PCR rounds, because each trypanosome species will generate a DNA fragment with a specific molecular weight. As multiple infections are common in tsetse flies and mammals, this technique appears, therefore, very useful in large scale studies aiming to identify natural trypanosome infections. Despite the low sensitivity of ITS, this technique enables the identification of several trypanosomes species; allowing thus a reduction of the analysis costs. With this technique, a considerable number of samples can be analyzed in a reasonable time and different trypanosome infections can be revealed.

The low infection rate of *T. brucei* s.l. in tsetse flies of the Malanga focus is in line with results obtained by Makumyaviri et al. [22] who identified very few infections of this trypanosome in domestic animals of the same area more than twenty years ago. Eleven of the 13 *T. brucei* infections were due to *T. b. gambiense,* suggesting that most of the *T. brucei* s.l. infections found in this area were due to *T. b. gambiense*. These results indicate a current circulation of *T. b. gambiense* in the HAT focus of Malanga. Our results suggest very low transmission of *T. b. brucei* in this focus because only 8 tsetse flies were found with this infection. These results are in line with those obtained in animals where only one domestic animal was found infected with this parasite [22]. The rarity of *T. b. brucei* in this zone can probably be explained by the fact that wild animals, known as being the reservoir of these parasites as well as the source of new infections, are scarce in this focus [22].

The identification of mixed infections involving different trypanosome species confirms previous results [6,23-27], and reflects either the prevalence of such infections in vertebrate hosts of this area, or frequent feeding of tsetse flies on animals infected with different trypanosome species. However, the low percentage (7.02%) of mixed infections involving different trypanosome species does not corroborate results obtained in other African regions where a high prevalence of mixed infections has been reported in tsetse flies [6,23-28]. The discrepancy between the percentages of mixed infections involving different trypanosome species can be explained by the availability of a suitable host for each trypanosome species. Despite the fact that the factors determining the distribution and abundance of trypanosomes are poorly known, it is likely, that most of these factors include the availability of suitable mammalian hosts [23]. The presence of mixed infections of *T. b. brucei* and *T. b. gambiense* confirms results obtained in Cameroon where about 69% of *T. b. gambiense* mid-gut infections were mixed with *T. b. brucei*[29].

Among the trypanosomes of the subgenus *Nannomonas*, *T. congolense* was the most common species found, and *T. congolense* savannah type the most prevalent. These results are in line with those reported in Southern Africa [23]. The high infection rate of trypanosomes belonging to *Nannomonas*, especially *T. congolense,* corroborates results obtained in tsetse flies of West, Central [25] and East Africa [23]. This is in line with the results obtained in domestic animals of the same area [22]. The high prevalence of *T. congolense* savannah type can be explained by geographical localization of the study area, which is located in the savannah zone.

The identification of different species of trypanosomes in tsetse of the Malanga HAT focus indicates the presence of trypanosomes causing HAT and AAT. Although the presence of *T. b. gambiense* in tsetse mid-guts is not proof of mature infection, since some mid-gut infections do not develop to maturation in the salivary glands, the identification of this parasite as well as the human blood meals in tsetse mid-guts indicates the circulation of *T. b. gambiense* between tsetse flies and man. This suggests an active transmission of HAT in the Malanga focus. Our results suggest that a flare up of the disease can occur at any time if control strategies are not put in place. This is of crucial importance for the HAT control program, especially at this moment in time, where active case detection is scarce in foci presenting low disease prevalence. The considerable number of pig blood meals associated with the identification of *T. b. gambiense* in tsetse mid-guts suggests that investigations on the animal reservoir of HAT at Malanga should be carried out.

## Conclusion

The results of this study (blood meal origins and different trypanosome species) have provided data that may help in the control and the surveillance of HAT and AAT. They may help to localize areas with higher transmission risk where vector control could be focused. If such control is undertaken, a direct effect will be observed on the animal reservoirs as well as on the number of infected flies. These approaches may be useful, especially currently, where the elimination of the disease has been envisaged in most HAT foci.

## Competing interests

The authors declare that they have no competing interests.

## Authors’ contributions

GS participated to the conception, the design of the study, the tsetse fly sampling and the drafting of the manuscript. BS was involved in the identification of blood meals as well as different species of trypanosomes. FN was involved in the conception of the study and the drafting of the manuscript. PL participated to the conception and the tsetse fly sampling. PM participated to the tsetse fly sampling. JM participated to the tsetse fly sampling. EZ participated to the tsetse fly sampling. RDD participated to the design of the study, the conception of pictures and the drafting of the manuscript. TA was involved in the conception of the study and the drafting of the manuscript. All authors read and approved the final version of the manuscript.
